# Effect of an algae feed additive on reducing enteric methane emissions from cattle

**DOI:** 10.1093/tas/txae109

**Published:** 2024-08-01

**Authors:** Reba L Colin, Jessica L Sperber, Kassidy K Buse, Paul J Kononoff, Andrea K Watson, Galen E Erickson

**Affiliations:** Department of Animal Science, University of Nebraska, Lincoln, NE 68583-0908, USA; Department of Animal Science, University of Nebraska, Lincoln, NE 68583-0908, USA; Department of Animal Science, University of Nebraska, Lincoln, NE 68583-0908, USA; Department of Animal Science, University of Nebraska, Lincoln, NE 68583-0908, USA; Department of Animal Science, University of Nebraska, Lincoln, NE 68583-0908, USA; Department of Animal Science, University of Nebraska, Lincoln, NE 68583-0908, USA

**Keywords:** algae, bromoform, carbon dioxide, emissions, enteric, methane

## Abstract

Alga 1.0, a product containing bromoform, was fed to cattle to evaluate its effects on methane (**CH**_**4**_) and carbon dioxide (**CO**_**2**_) emissions and diet digestibility. Twelve nonlactating, nonpregnant Jersey cows (490 ± 19 kg body weight) were used in four replicated 3 × 3 Latin squares with three periods, each consisting of 21 d. Cows were blocked by feed intake (averaged intakes over 4 wk prior to trial) and assigned randomly to one of three treatments. Treatments included Alga 1.0 fed at 0, 69, and 103 g/d in a 0.454 kg/d dry matter (**DM**) top-dress daily in a modified distillers grains plus solubles (**MDGS**) carrier. Diet consisted of 60% dry-rolled corn, 20% corn silage, 15% modified distillers grains, and 5% supplement (DM basis). Headbox-style indirect calorimeters were utilized to evaluate gas production from individual cows with two nonconsecutive 23-h collections in each period. Data were analyzed using the GLIMMIX procedure of SAS with cow within square as experimental unit and as a random effect, and treatment and period as fixed effects. Linear and quadratic contrasts were used to compare treatments. Feeding Alga 1.0 linearly reduced dry matter intake (**DMI**, *P* < 0.01) by 10.1% for 69 g/d inclusion and 13.3% for 103 g/d inclusion compared to the control. Nutrient intakes decreased linearly (*P* < 0.01) due to lower DMI, but nutrient digestibility was not impacted (*P* ≥ 0.28). Inclusion of Alga 1.0 did not impact gross energy or digestible energy concentration of the diets expressed as Mcal/kg DM (*P* ≥ 0.22) but did linearly reduce energy intake (Mcal/d; *P* < 0.01). Feeding Alga 1.0 linearly reduced enteric CH_4_ emissions measured as g/kg DMI (*P* < 0.01) by 39 and 64% for 69 g/d and 103 g/d inclusion, respectively. Linear reductions (*P* < 0.01) of 64% to 65% were also observed in enteric CH_4_ emissions when expressed per kilogram of DM or organic matter digested. Respired CO_2_ as g/d linearly decreased (*P* = 0.03) for cattle fed Alga 1.0 but did not differ when expressed as g/kg of DMI (*P* ≥ 0.23). Oxygen consumption did not differ between treatments for g/d and g/kg DMI (*P *≥ 0.19). In conclusion, feeding Alga 1.0 reduced DMI up to 13.3%, did not impact digestibility, and significantly reduced CH_4_ emissions up to 63%.

## Teaser

Feeding increasing inclusions of Alga 1.0 (bromoform-containing algae product) reduced enteric methane production up to 65% in cattle fed a finishing diet.

## INTRODUCTION

Greenhouse gas (**GHG**) emissions are a concern related to present and future climate and global warming potential. Methane (**CH**_**4**_), produced via enteric fermentation, is a GHG of interest to the agricultural industry due to its climate warming potential. Since the half-life of CH_4_ is relatively short, spending an average of 10 to 12 yr in the atmosphere, reducing CH_4_ emissions will have a positive short-term impact on climate (Allen, 2016). Reducing CH_4_ production from ruminants can be beneficial for the environment by lessening the emission impact on global warming, reducing the impact of animal production on GHG emissions, and using GWP* suggests reduction of CH_4_ by cattle would have rapid impacts.

As of recent, there has been interest in using algae as a strategy to reduce enteric CH_4_ emissions from ruminants (Roque et al., 2019, 2021). Algae include a broad category of aquatic plants, including macroalgae, which can grow in fresh or salt water and vary greatly in chemical makeup among different types, such as being rich in protein or lipids (Makkar et al., 2016). Red algae (*Rhodophyceae*) is the primary algae type reported for significant reduction in enteric CH_4_ emission due to high concentrations of haloforms (Machado et al., 2016), with the *Asparagopsis taxiformis* species resulting in a reduction of up to 80% for enteric CH_4_ emissions for cattle consuming a low-forage diet (Roque et al., 2021).

Certain algae types have concentrated quantities of bromoform, the active metabolite that blocks the pathway for CH_4_ production during fermentation by rumen microbes (Machado et al., 2016). Bromoform blocks key metalloenzymes via the Wolfe cycle (Glasson et al., 2022), and when this pathway is blocked, methyl transfer of the cobamide-dependent enzyme needed to catalyze the final step for methanogenesis is inhibited (Fouts et al., 2022; Glasson et al., 2022). The inhibition of the CH_4_ production cycle is effective in reducing methane emissions. While bromoform is implicated in ozone depletion, the potential to impact the ozone layer is minimal (Jia et al., 2022). With proper handling of bromoform, researchers believe it can be utilized as a feed additive that effectively reduces CH_4_ production from ruminants (Fouts et al., 2022). Bromoform has been identified as a potential carcinogen (Glasson et al., 2022) so it is important to note that not all algae strains are FDA-approved for feeding to cattle, and products fed to cattle that enter into the human food supply are required to meet stringent safety measures. The algae evaluated in the current experiment is trade-named Alga Bio 1.0 (Alga Biosciences, Berkeley, CA) and is a proprietary feed additive containing stabilized bromoform in conjunction with generally recognized as safe ingredients.

The objective of this experiment was to evaluate the effect of Alga 1.0, an algae-derived manufactured product containing bromoform, on methane production and diet digestibility. It was hypothesized that increasing inclusions of Alga 1.0 would further reduce enteric CH_4_ production from cattle without negatively affecting diet digestibility.

## MATERIALS AND METHODS

### Animals and Treatments

Animal care and experimental procedures were approved by the University of Nebraska-Lincoln Animal Care and Use Committee (Protocol 2188). Twelve nonlactating, nonpregnant Jersey cows (490 ± 19 kg body weight [**BW**]) were used to determine the effect of Alga 1.0 (Alga Biosciences) inclusion on the production of enteric CH_4_ and CO_2_ emissions. The Jersey cows utilized in this experiment were previously trained for emission monitoring via headbox which alleviated the need for an additional training period for new cattle. In addition, cattle fed this product would not be allowed to enter the food chain so were suitable to use for this study. The cows were nonlactating and of a type where intake would mimic beef systems. Cows were housed in individual tie-stalls in an environmentally controlled area at the Dairy Metabolism Facility in the Animal Science Complex at the University of Nebraska-Lincoln (Lincoln, NE). Since the Alga 1.0 product was not approved for feeding to cattle at the time of this experiment, no meat from these animals entered the food supply chain and cows were euthanized and incinerated upon experiment completion.

The experimental design was a replicated 3 × 3 period Latin square with four squares, with three periods each lasting 21 d. This experiment was conducted with all 12 cows collected simultaneously. Prior to experiment initiation, nonlactating cows were grouped by dry matter intake (DMI), calculated from the average of previous intake over a 4-wk period. Treatments included 0 g (negative control), 69 g, and 103 g (DM basis) of Alga 1.0 fed daily. This was equal to approximately 0% (negative control), 0.77%, and 1.2% of diet DM and 0%, 0.8%, and 1.3% of diet OM. Modified distillers grains plus solubles (**MDGS**) was used as the carrier due to the palatability of MDGS and was delivered via top-dress on the ration at 0.454 kg DM daily. This approach was done to avoid any cross-contamination of the feed mixer used to deliver feed to all cows. In addition, top-dressing allowed for a targeted amount to be delivered daily. Prior to experiment initiation, cows were transitioned to a basal diet by feeding three intermediate diets (grass hay replaced by corn in each subsequent diet) fed for 7 d each. The basal diet consisted of 60% dry-rolled corn (**DRC**), 20% corn silage, 15% MDGS, and 5% supplement (DM basis; Table 1). Dietary ingredients for the primary diet (corn silage, MDGS, DRC, and supplement) were added to a Calan Data Ranger (American Calan, Inc., Northwood, NH), mixed, and fed as a total mixed ration (**TMR**) once daily with a target refusal rate of 5%. The top-dress containing Alga 1.0 was hand-mixed and added immediately after TMR delivery; where a small indentation was formed in the feed delivery, in which top-dress was placed to reduce the cattle’s ability to sort. Each period included 16 d of ad libitum diet adaptation, followed by 4 d of total collection where diets were fed at 100% of the previous 6 d intake to limit refusal.

**Table 1.** Ingredient and nutrient composition of finishing diet including 0, 69, or 103 g/d of Alga 1.0 fed to Jersey cows

Ingredient	Percent of Diet DM^1^
Dry-rolled corn	60
Corn silage	20
MDGS^2^	15
Supplement^3^	5
Fine ground corn	2.203
Limestone	1.68
Tallow	0.125
Urea	0.60
Salt	0.30
Trace mineral premix	0.05
Vitamin ADE	0.015
Rumensin-90^4^	0.0165
Tylan-40^5^	0.011

^1^All treatments received the same basal diet with the addition of Alga 1.0 as a top-dress (0, 69, or 103 g/d) mixed with MDGS at 0.454 kg DM/cow daily.  The Alga 1.0 inclusion was approximately 0.77% and 1.2% of diet DM for 69 and 103 g/d consumption on average, respectively.

^2^MDGS, modified distillers grains plus solubles.

^3^Supplement fed at 5% of dietary DM.

^4^Formulated to provide monensin (Rumensin-90; Elanco Animal Health; Greenfield, IN) at 33.7 mg/kg DM basis.

^5^Formulated to supply 9.7 mg/kg tylosin phosphate (Tylan; Elanco Animal Health; DM basis).

### Sample Collection and Analysis

All samples and data collection were performed according to McLain et al. (2021). Body Condition Score (**BCS**) was measured using a 5-point scale common to the U.S. dairy industry (Roche et al., 2004). Individual feed ingredients and TMR were sampled daily during each collection period and frozen at −20 °C. Feed ingredients were dried at 60 °C for 48 h and ground through a 1-mm screen (Wiley Mill; Arthur A. Thomas Co., Philadelphia, PA). A subsample of ground feed was sent to Cumberland Valley Analytical Services Inc. (Waynesboro, PA) for analysis of starch (Hall, 2009), crude protein (**CP**; method 990.03, AOAC, 2000), neutral detergent fiber with sodium sulfite and α amylase corrected for ash contamination (**aNDFom**) (Van Soest, et. al., 1991), acid detergent fiber (**ADF**; method 973.18, AOAC, 2000) modified to use Whatman 1.5-µm microfiber filters, and ash (method 942.05, AOAC, 2000). Additionally, feed ingredients were analyzed for dry matter (**DM**), organic matter (**OM**), and gross energy (**GE**) content (Parr 6400 Calorimeter, Moline, IL) in the nutrition laboratory at the University of Nebraska-Lincoln. Samples were dried at 100 °C for 24 h to determine DM and then burned in a cool muffle furnace at 600 °C for 6 h to determine OM (method 942.05, AOAC, 2000). During each day of the collection period, refusals were weighed, sampled, and composited. Refusals were analyzed for DM, OM, GE, CP, starch, and aNDFom via the same methods as described for feed ingredients.

Total fecal output was collected from each individual cow during the collection period for four consecutive days. A rubber mat was placed behind each cow to aid in fecal collection, in which feces were manually collected by personnel during defecation or picked up from the rubber mat and deposited into a rubber trash can. A trash bag was placed over the trash can to minimize nitrogen losses prior to subsampling. Urine contamination was eliminated by inserting a 30 French foley catheter (BARD, Covington, GA) into each cow’s bladder with a stylus. The balloon was inflated to 60 mL with physiological saline. The catheter was drained into a drainage grate using plastic Tygon tubing. Metabolizable energy was not calculated as the urine was discarded (not quantified or sampled). Daily feces were weighed, subsampled, composited on a weight basis (approximately 10% of total weight), and frozen between collection events. After collections, feces were dried at 60 °C for 48 h and ground through a 1-mm screen (Wiley Mill, Arthur H. Thomas Co., Philadelphia, PA). The ground feces samples were analyzed for DM, OM, GE, CP, starch, and aNDFom as described for feed ingredients.

### Gas Collection

Gas emissions from individual cows were determined via headbox-type indirect calorimeters built at the University of Nebraska-Lincoln and described by McLain et al. (2021). For each cow, a collection period of two nonconsecutive, 23-h periods during the last 5 d of the period were used to measure CO_2_ and CH_4_ production. The collection method was similar to that described by Foth et. al. (2015). Gas production was adjusted to represent a 24-h period. Feed was placed in the bottom of the headbox, and cows were allowed ad libitum access to water from a water bowl located inside the headbox. Feed was placed in the headbox when the cows entered at 0930 hours.

The doors of the headbox were closed after feeding and the vacuum motor (Model 115923, Ametek Lamb Electric, Kent, OH) was turned on, which created a negative pressure system inside the headbox. Temperature and moisture within the headbox were measured every minute using a probe (Model TRH-100, Pace Scientific Inc., Moorseville, NC) and recorded using a data logger (Model XR440, Pace Scientific Inc.). Barometric pressure of the room was measured using a barometer (Chaney Instruments Co.) and recorded hourly by personnel. The total volume of gas that flowed through the headbox was measured using a gas meter (Model AL425, American Meter, Horsham, PA) and corrected to standard temperature and pressure (0 °C, 760 mmHG) with adjustment for moisture content of exhaust air. Ambient air and air within the headbox were collected into separate bags (44 L, LAM-JAPCON-NSE; Pollution Measurement Corp., Oak Park, IL). Gas bags were analyzed for oxygen (**O**_**2**_), CO_2_, and CH_4_ using an Emerson X-stream 3-channel analyzer (Solon, OH) according to the method of Nienaber and Maddy (1985). Gas measurements collected over the 2 d were averaged to obtain one value per period per cow. During collections, a 4 d DMI average was used to obtain one value per period per cow to quantify gas produced on a DMI basis.

### Energy Calculations

The respiratory quotient (**RQ**) was calculated using the ratio of CO_2_ produced to O_2_ consumed (L/L). Digestible energy (**DE**) was calculated as follows:


$$\begin{array}{l} DE(Mcal/d)=GE(Mcal/d)fecal\;energy(Mcal/d) \end{array}$$

### Statistical Analysis

Data were collected and stored using a Microsoft Excel spreadsheet (Microsoft Corporation, Redmond, WA) followed by analysis using the GLIMMIX procedure of SAS 9.4 (SAS Inst. Inc., Cary, NC). All data collected were analyzed for statistical outliers (more than 2.5 standard deviations from the mean), but no observations were identified or removed from the analysis. A Latin square design was utilized with cow within square as the experimental unit included as a random effect. The model included treatment and period as fixed effects. Linear and quadratic contrasts were analyzed using the GLIMMIX procedure of SAS subsequent to using Proc IML to determine the coefficients used for contrasts due to uneven spacing of treatments. Significance was considered at α ≤ 0.05 and a tendency was considered at 0.05 < α ≤ 0.10.

## RESULTS AND DISCUSSION

### Intake and Digestibility

DMI (kg/d) was linearly reduced with the inclusion of Alga 1.0 (*P *< 0.01) compared to the dry-rolled corn control diet (Table 2). There was a 10.1% and 13.3%, or 1.01 and 1.32 kg, reduction in DMI with 69 and 103  inclusion of Alga 1.0, respectively. Roque et al. (2019) included *A. armata* in lactating dairy cow diets, reporting DMI reductions of 10.8% and 38.0% for 0.5% and 1.0% OM inclusion of algae, respectively, compared to DMI reductions of 10.1% and 13.3% in the current experiment with algae inclusion of 0.8% and 1.3% OM intake of Alga 1.0, respectively. The reduced intake in this study cannot be due to digestibility changes or passage as gut fill is not a limitation to DMI with the grain-based diet fed. The product could be less palatable although supplementation was top-dressed so should not depress overall intake and cattle readily consumed the top-dressed supplement. There may be disruptions to fermentation due to CH_4_ inhibition which is the most likely cause of depressed DMI as Algae 1.0 increased, although that response has not been consistent across studies.

**Table 2.** Effect of Alga 1.0 inclusion on intake and digestibility when fed at 0, 69, or 103 g/d to Jersey cows fed a feedlot finishing diet

Intake and Digestibility	Treatment^1^			SEM	Contrasts	
	Control	69 g	103 g		Linear	Quadratic
DM						
Intake, kg/d	9.96	8.95	8.64	0.64	<0.01	0.74
Digestibility, %	71.3	69.7	69.5	1.77	0.28	0.83
OM						
Intake, kg/d	9.47	8.49	8.20	0.606	<0.01	0.71
Digestibility, %	75.8	75.2	75.3	1.31	0.70	0.82
CP						
Intake, kg/d	1.36	1.24	1.19	0.089	<0.01	0.92
Digestibility, %	70.5	70.8	70.3	0.92	0.94	0.76
Starch						
Intake, kg/d	5.09	4.53	4.41	0.32	<0.01	0.57
Digestibility, %	91.7	92.4	92.6	1.22	0.50	0.91
aNDFom						
Intake, kg/d	1.71	1.56	1.48	0.112	<0.01	0.95
Digestibility, %	39.9	36.8	37.7	3.03	0.40	0.56

^1^All treatments received the same basal diet with the addition of Alga 1.0 as a top-dress (0, 69, or 103 g/d) mixed with modified distillers grains plus solubles at 0.454 kg DM/cow daily. The Alga 1.0 inclusion was approximately 0.77% and 1.2% of diet DM for 69 and 103 g/d inclusions, respectively.

Kinley et al. (2020) fed *A. taxiformis* in feedlot diets at 0%, 0.05%, 0.10%, and 0.20% OM inclusion, reporting an increase in DMI between the 0.05% and 0.10% OM inclusion of algae, but no differences in DMI between the other treatment inclusion rates. Roque et al. (2021) compared *A. taxiformis* (seaweed) inclusion rates at 0%, 0.25%, and 0.50% of OM in a high-forage feedyard starter diet (60.0% forage + 20.0% DRC), medium-forage feedyard transition diet (40.0% forage + 37.0% DRC), and low-forage feedyard finisher diet (11.0% forage + 72.0% DRC). Roque et al. (2021) reported no differences in DMI for differing seaweed inclusions in the high-forage diet and low-forage diet, however, when steers were fed a medium-forage diet (40.0% forage), the 0.25% and 0.50% OM seaweed inclusion reduced DMI by 1.4 and 2.2 kg/d, respectively. In addition, Roque et al. (2021) analyzed the impact of seaweed inclusion on DMI over the entire feeding period, reporting a tendency for 8.0% reduction in DMI for 0.25% OM seaweed inclusion, and 14.0% reduction in DMI for 0.50% OM seaweed inclusion. Dissimilar to results reported by Kinley et al. (2020) and Roque et al. (2021), a meta-analysis evaluating the effect of algae on enteric CH_4_ emission by Lean et al. (2021) reported no effect of algae inclusion in the diet on DMI in dairy and beef cattle.

Dry matter digestibility was not impacted (*P* ≥ 0.28) by the inclusion of Alga 1.0 in the current experiment and averaged 70.2% across the three treatment inclusion levels (Table 2). There was a 2% unit decrease (71.3% to 69.5% DM digestibility) as Algae 1.0 increased which is likely due to increased minerals in the Algae treatments that are not digested as OM digestibility was not impacted statistically (*P* ≥ 0.70) and were almost identical across treatments. Krizsan et al. (2023) reported similar results where DM digestibility did not differ with the inclusion of *A. taxiformis* at 0.50% of OM intake in a grass silage diet. Stefenoni et al. (2021) compared varying inclusions at 0%, 0.25%, and 0.50% DM *A. taxiformis*, reporting a quadratic effect on DM digestibility with 61.7% and 65.2% DM digestibility for 0.25% and 0.50% DM, respectively, and reported no difference for 0% and 0.50% inclusion levels.

Similar to the linear reduction observed for DMI (kg/d), OM, starch, aNDFom, and CP intake (kg/d) linearly decreased (*P *≤ 0.01) as Alga 1.0 inclusion increased in the diet (Table 2) which is driven by DM intake as the composition of diets were similar across treatments. All nutrient intakes reported by Stefenoni et al. (2021) were similar to the current experiment; however, they utilized dairy cows consuming a high-forage (60%) diet compared to the high-concentrate diet utilized in this experiment. Krizsan et al. (2023) utilized *A.taxiformis* in a grass silage diet fed to dairy cows and reported similar reductions in OM and NDF intakes. Digestibility of OM, CP, starch, and aNDFom were not significantly affected by the inclusion of Alga 1.0 (*P* ≥ 0.40). Dissimilar to the results from this experiment, Stefenoni et al. (2021) reported a significant increase in OM digestibility when *A. taxiformis* was increased in the diet from 0.25% to 0.50%, although there was no difference observed between the control and 0.50%. Krizsan et al. (2023) reported that the inclusion of *A. taxiformis* at 0.50% of diet OM had no effect on the digestibility of OM or NDF.

### Energy and Performance

The **GE** provided from Alga 1.0 was 3.32 Mcal/kg (Table 3) and was similar to previous literature in which algae GE was evaluated (Makkar et al., 2016). The GE intake of the diet, reported as Mcal/d, linearly decreased (*P* < 0.01), up to 5.3 Mcal/d or 13.5%, with the increasing inclusion of Alga 1.0 (Table 4). However, GE concentration expressed as Mcal/ kg of DM was not different (*P* ≥ 0.22) between treatments. This trend followed for DE where Mcal/d linearly reduced (*P* < 0.01) by 10.6% and 14.5% for 69 and 103 g algae inclusion, respectively; however, no significant difference was observed for DE (*P *≥ 0.38) when expressed as Mcal/kg. The ratio of DE/GE was not different (*P *≥ 0.37) between treatments where the control measured 0.72 and the Alga 1.0 treatments measured 0.71.

**Table 3.** Chemical composition of diet ingredients^1^ and Alga 1.0 fed at 0, 69, or 103 g/d to Jersey cows fed a feedlot finishing diet

	Dry-rolled corn	Corn silage	MDGS^2^	Supplement	Alga 1.0^3^
Dry matter, % as is	87.5	37.0	50.6	92.7	87.4
Organic matter	98.4	95.5	97.8	61.5	81.3
Crude protein	8.60	9.20	33.6	37.2	7.40
aNDFom^4^	9.67	33.1	30.9	8.13	20.9
ADF^5^	3.90	21.2	9.67	4.30	1.47
Starch	68.4	40.2	2.00	26.3	4.73
Gross energy, Mcal/kg	4.07	4.11	4.62	1.98	3.32

^1^Average composition for three samples of DRC, corn silage, MDGS, supplement, and Alga 1.0 analyzed at Cumberland Valley Analytical Services Inc. (Waynesboro, PA) for all nutrients except GE analyzed at UNL using bomb calorimetry.

^2^MDGS = Modified distillers grains plus solubles.

^3^Alga 1.0 = Proprietary algae feed additive (Alga Biosciences, Berkeley, CA) containing stabilized bromoform.

^4^Adjusted neutral detergent fiber corrected for ash contamination.

^5^Acid detergent fiber.

**Table 4.** Effect of Alga 1.0 when fed at 0, 69, or 103 g/d to Jersey cows fed a feedlot finishing diet on cow performance and energy

	Treatment^1^			SEM	Contrasts	
	Control	69 g	103 g		Linear	Quadratic
Gross energy, Mcal/d	39.3	35.3	34.0	2.59	<0.01	0.77
Gross energy, Mcal/kg DM	4.04	4.05	4.04	0.004	0.99	0.22
Digestible energy, Mcal/d	28.3	25.2	24.1	1.90	<0.01	0.82
Digestible energy, Mcal/kg DM	2.93	2.89	2.87	0.056	0.33	0.99
DE/GE^2^	72.3	71.3	71.0	1.40	0.36	0.95

^1^All treatments received the same basal diet with the addition of Alga 1.0 as a top-dress (0, 69, or 103 g/d) mixed with modified distillers grains plus solubles at 0.454 kg DM/cow daily. The Alga 1.0 inclusion was approximately 0.77% and 1.2% of diet DM for 0, 69, and 103 g/d inclusions, respectively.

^2^DE/GE, digestible energy (Mcal/d)/gross energy (Mcal/d) × 100.

BW and BCS of the cow were not impacted (*P* ≥ 0.17) by the inclusion of Alga 1.0 (data not shown). Average BW during the study was 497 kg and BCS averaged 3.87. Both increased across periods (time of study) but the study was not designed to assess the impact of treatment on BW due to short period durations. This outcome in BW matches observations reported by Roque et al. (2019), who reported no differences in BW of dairy cows when including *A. armata* at varying inclusions in the diet over 21-d periods. Both Roque et al. (2019) and the current experiment were designed as 3 × 3 Latin squares with 21-d periods, therefore, BW and BCS differences between treatments over a 21-d period would not be expected.

### Gas Production

The inclusion of Alga 1.0 in the diet linearly reduced enteric CH_4_ emission measured as g/d (*P* < 0.01; Table 5) and g/kg DMI (*P* < 0.01). Alga 1.0 inclusion reduced enteric CH_4_ emission by 46% or 76.7 g/d when fed at 69 g/d and 73% or 120.6 g/d when fed at 103 g/d compared to the negative control. Methane yield (g/kg DMI) decreased 6.7 and 10.9 g/kg DMI with the inclusion of Alga 1.0 at 69 and 103 g, respectively, accounting for a 39% reduction in CH_4_ emissions expressed as g/kg of DMI with 69 g/d Alga 1.0 and 64% reduction (g/kg of DMI) with 103 g/d Alga 1.0 compared to the negative control. These results indicate that feeding Alga 1.0 reduces CH_4_ emissions from cattle consuming feedlot diets typical to the U.S. Results from this experiment are similar to previous literature, where increasing levels of bromoform-containing algae reduced CH_4_ emissions (Roque et al., 2019; Kinley et al., 2020). Roque et al. (2019) included *A. armata* algae at 0.5% and 1.0% OM in a high-forage diet common to the U.S. dairy industry, reporting a 26.4% and 67.2% reduction in CH_4_ production (g/d) and 20.5% and 42.6% reduction in CH_4_ production expressed as g/kg of DMI for 0.5% and 1.0% OM inclusion, respectively. The overall reduction in CH_4_ production for the current experiment was similar to CH_4_ reductions reported by Roque et al. (2019), however, the algae inclusion levels for the current experiment were slightly greater at 0.8% and 1.3% of OM. Similar responses were observed when evaluating CH_4_ emissions as a proportion of DM and OM digestion. Expressed as g/kg of DM digestion, CH_4_ emissions decreased linearly (*P* < 0.01) by 63.7% when Alga 1.0 was increased. Very similar linear (*P* < 0.01) reductions of 65% for CH_4_ emissions expressed as g/kg of OM digested were observed as Alga 1.0 increased. These data suggest that CH_4_ emissions reduction due to Alga 1.0 are independent of lower intake as CH_4_ emissions expressed as g/kg of DMI decreased 64%. Additionally, observing a 65% reduction in CH_4_ emissions when expressed as g/kg of OM digested suggested energy available to cattle would lead to reductions of CH_4_ emissions when expressed as intensity (g/kg of gain) although gain was not adequately addressed with this experimental design.

**Table 5.** Effect of Alga 1.0 when fed at 0, 69, or 103 g/d to Jersey cows fed a feedlot finishing diet on gaseous loss and gaseous consumption

	Treatment^1^			SEM	Contrasts	
	Control	69 g	103 g		Linear	Quadratic
CH_4_, g/d	164.9	88.1	44.3	13.8	<0.01	0.81
CH_4_, g/kg DMI	17.0	10.3	6.1	1.62	<0.01	0.65
CH_4_, g/kg DM digested	23.7	14.9	8.6	2.25	<0.01	0.52
CH_4_, g/kg OM digested	23.5	14.4	8.3	2.17	<0.01	0.56
CO_2_, g/d	8420	7835	7719	419.5	0.03	0.68
CO_2_, g/kg DMI	873	891	914	40.9	0.23	0.75
O_2_ consumption, g/d	5729	5025	5390	368.1	0.26	0.19
O_2_ consumption, g/kg DMI	603	564	628	43.0	0.78	0.21
RQ^2^	1.04	1.02	1.02	0.012	0.17	0.50

^1^All treatments received the same basal diet with the addition of Alga 1.0 as a top-dress (0, 69, or 103 g/d) mixed with modified distillers grains plus solubles at 0.454 kg DM/cow daily. The Alga 1.0 inclusion was approximately 0.77% and 1.2% of diet DM for 0, 69, and 103 g/d inclusions, respectively.

^2^RQ, respiratory quotient, liters per day of CO_2_ production/liters per day of O_2_ consumption.

Kinley et al. (2020) observed reductions of 9.0%, 38.0%, and 98.0% for CH_4_ production (g/kg DMI) with incremental inclusions of *A. taxiformis* at 0.05%, 0.10%, and 0.20% OM, respectively. Roque et al. (2021) fed *A. taxiformis* at 0.25% and 0.50% OM inclusion and observed a 69.8% and 79.8% reduction in CH_4_ production (g/kg DMI). The ability of a seaweed to reduce enteric CH_4_ production when fed in ruminant diets is largely dependent on bromoform concentration in the diet. When comparing Kinley et al. (2020), Roque et al. (2021), and the current experiment, although the reductions of CH_4_ production were not consistently linear with increasing inclusion of bromoform in the diet, all three experiments reported dose-dependent reductions in CH_4_, where the greatest inclusion of bromoform resulted in the greatest reduction in methane.

Bromoform is a volatile compound that can lose potency when exposed to improper handling/holding temperature and airflow. A loss in bromoform potency may result in reduced ability of the product to alter ruminal CH_4_ production. Proper storage, handling, and shelf-life understanding of algae is critical when considering the efficacy of the product as an enteric methane mitigation strategy. Stefenoni et al. (2021) evaluated the impacts of storage length and method on bromoform concentration in algae, reporting up to 85% reduction in bromoform concentration when stored for long periods of time (4 mo or greater) and exposed to air and improper temperature. In the current experiment, Alga 1.0 product was frozen at −4 °C in dark conditions. Alga 1.0 product was mixed with MDGS and placed in airtight bags, and subsequently fed to cattle within 3 d of mixing. A potential loss of bromoform concentration and therefore, reduced potential for CH_4_ reduction, may have been due to the dietary intake pattern variation between cows, where some cattle would require longer lengths of time throughout the day to consume their allotted feed, subsequently increasing time that Alga 1.0 was exposed to air and allowing for bromoform volatilization. Increased bromoform volatilization may result in a reduced impact on enteric CH_4_ emission, as there would be reduced active bromoform ingredients in the feed additive.

Production of CO_2_ reported in g/d linearly decreased (*P* = 0.03) up to 585 g/d or 6.9% for cattle receiving 69 g/d and by 8.3% or 701 g/d for cattle fed 103 g/d of Alga 1.0, however, CO_2_ did not differ between treatments (*P* = 0.23) when normalized for the amount of feed intake (calculated as g/kg DMI; Table 5). These results differ from CO_2_ production and yield reported by Roque et al. (2021), who observed no difference in g/d of CO_2_ but an increase in CO_2_ g/kg of DMI when comparing 0.50% OM inclusion of macroalgae (*A. taxiformis*) compared to a negative control. Roque et al. (2019) reported a decrease in CO_2_ (g/d) of 13.9% when comparing cattle fed a macroalgae (*A. Armata)* at 1.0% inclusion compared to a negative control, similar to what was observed in the current experiment; however, they did observe a significant increase in CO_2_ when normalized for amount of feed intake (g/kg of DMI) with increasing inclusions of *A. armata*. Oxygen consumption amounts did not differ between treatments when measured as g/d (*P* ≥ 0.19) and g/kg DMI (*P* ≥ 0.21). RQ (a measure of basal metabolic rate) was not significantly impacted (*P* ≥ 0.17) by treatments and averaged 1.03. The energy density provided to cattle on a feedlot diet is high, and therefore, a high RQ is expected as the cattle are not catabolizing body tissue for energy, where an RQ of one indicates that carbohydrates are the primary source of energy and that the animals are likely to accumulate lipids (Forbes et al., 1927). Therefore, the RQ values observed in the current experiment were what is expected for cattle consuming a high-concentrate feedlot diet.

## IMPLICATIONS

Cattle fed a finishing diet supplemented at 69 or 103 g/d with this proprietary algae product containing bromoform had reduced CH_4_ emissions compared to cattle that were not supplemented. Reductions of 39% and 63% were observed for CH_4_ yield (g/kg DMI) with the inclusion at 69 and 103 g/d, respectively. Nutrient intakes were reduced due to DMI being reduced by 10.1% and 13.3% with the increasing inclusion of Alga 1.0 in the diet, however, digestibility and dietary energy concentration of the diet were not impacted suggesting a 65% reduction in methane per unit of OM digested. Further research is needed to quantify the impact of this supplement on cattle performance. Feeding this product in a high-concentrate diet is an effective CH_4_ mitigation tool when fed to finishing cattle if appropriate regulatory approvals are obtained.
